# Scaling up kangaroo mother care in South Africa: 'on-site' versus 'off-site' educational facilitation

**DOI:** 10.1186/1478-4491-6-13

**Published:** 2008-07-23

**Authors:** Anne-Marie Bergh, Elise van Rooyen, Robert C Pattinson

**Affiliations:** 1 MRC Research Unit for Maternal and Infant Health Care Strategies, South Africa; 2 Department of Obstetrics and Gynaecology, University of Pretoria, South Africa; 3 Department of Paediatrics, University of Pretoria, South Africa

## Abstract

**Background:**

Scaling up the implementation of new health care interventions can be challenging and demand intensive training or retraining of health workers. This paper reports on the results of testing the effectiveness of two different kinds of face-to-face facilitation used in conjunction with a well-designed educational package in the scaling up of kangaroo mother care.

**Methods:**

Thirty-six hospitals in the Provinces of Gauteng and Mpumalanga in South Africa were targeted to implement kangaroo mother care and participated in the trial. The hospitals were paired with respect to their geographical location and annual number of births. One hospital in each pair was randomly allocated to receive either 'on-site' facilitation (Group A) or 'off-site' facilitation (Group B). Hospitals in Group A received two on-site visits, whereas delegates from hospitals in Group B attended one off-site, 'hands-on' workshop at a training hospital. All hospitals were evaluated during a site visit six to eight months after attending an introductory workshop and were scored by means of an existing progress-monitoring tool with a scoring scale of 0–30. Successful implementation was regarded as demonstrating evidence of practice (score >10) during the site visit.

**Results:**

There was no significant difference between the scores of Groups A and B (p = 0.633). Fifteen hospitals in Group A and 16 in Group B demonstrated evidence of practice. The median score for Group A was 16.52 (range 00.00–23.79) and that for Group B 14.76 (range 07.50–23.29).

**Conclusion:**

A previous trial illustrated that the implementation of a new health care intervention could be scaled up by using a carefully designed educational package, combined with face-to-face facilitation by respected resource persons. This study demonstrated that the site of facilitation, either on site or at a centre of excellence, did not influence the ability of a hospital to implement KMC. The choice of outreach strategy should be guided by local circumstances, cost and the availability of skilled facilitators.

## Background

Implementing and scaling up new health care interventions is very challenging and often demands intensive training or retraining, especially when the objective is to reach a health system on a provincial or national level. According to a systematic review of interventions by Grimshaw et al., the successful implementation of a programme depends, among others, on face-to-face communication, the use of a multimedia package for training, the development of protocols and guidelines within individual institutions, and opinion leaders at grassroots level who are convinced of the value of the programme [1]. As this is an expensive option in terms of human resources requirements for the introduction of new health care interventions, the South African Medical Research Council's (MRC) Research Unit for Maternal and Infant Health Care Strategies is involved in a long-term research programme to test the effectiveness of different outreach strategies for scaling up interventions or quality improvement programmes, some of which could potentially be more cost-efficient. This is being done in collaboration with different provincial and local health care authorities, and involves primary health care clinics, community health centres and hospitals. Four initiatives are currently under way – kangaroo mother care (KMC), basic antenatal care, basic intrapartum care and essential steps in postpartum care.

The focus of the kangaroo mother care initiative was to introduce KMC in all health care facilities in South Africa, starting with hospitals that provide newborn care, followed by home-based KMC in the community. KMC, the method of choice for hospitals caring for stable immature infants [2], is an alternative to conventional incubator and bassinet care. The infant is positioned skin-to-skin between the mother's breasts and secured firmly. KMC programmes also include the promotion of breastfeeding and the ambulatory support of mothers after discharge. The advantages and practice of KMC, even for unstable low birth-weight infants and healthy newborns, have been well documented and described in the literature [3-8].

The effectiveness of three different outreach strategies in provincial scale-up programmes has now been tested in South Africa, using KMC as the example of a new health care intervention. Although hospitals were used in this study, the principles are also applicable and the findings transferable to community-based interventions. In 2002, two strategies were tested as part of the Ukugona Outreach in the Province of KwaZulu-Natal. Hospitals were paired and assigned either to receiving an evidence-based multimedia educational package on its own or to receiving on-site regional facilitation in conjunction with the use of the package. The results of the study confirmed Grimshaw et al.'s observation [1] – facilitation using an on-site, face-to-face strategy, combined with a carefully designed implementation package, was found to be significantly more effective than using the package on its own [9].

When the MRC Unit was approached by the Ministries of Health of the Gauteng and Mpumalanga Provinces to assist with the implementation of KMC, the opportunity arose, for the first time, to test the effectiveness of two different outreach strategies using face-to-face facilitation. The two strategies were 'on-site', face-to-face facilitation at individual health care facilities (a strategy that had been demonstrated to be effective in the first trial) and 'off-site', face-to-face facilitation at a centre of excellence (the 'new' intervention). The design and results of this trial will be described in this paper.

### Implementation process

Ideally a new health care intervention should be introduced in all the relevant health care facilities simultaneously. This was the approach followed in the Ukugona Outreach [9]. However, practical constraints, budgetary considerations and the availability of human resources are realities that often have to be taken into account when planning an outreach. Both provinces participating in this study decided on a staggered approach, whereby a certain number of the targeted hospitals were included in the outreach each year. The Sub-directorate: Maternal, Child and Women's Health of the Gauteng Department of Health was responsible for the implementation of KMC in this province. They launched the Fara Ngwana ('hold the baby') outreach in August 2003. In the Mpumalanga Province the Ukubamba Umtwana Kuwe ('hold the baby tightly') outreach, launched in March 2004, was the responsibility of the Subdirectorate: Nutrition of the Department of Health and Social Services and was one of the priority programmes of the Integrated Nutrition Programme. In Gauteng seven hospitals were targeted for implementation support in 2003 and another five in 2005. In Mpumalanga seven hospitals were targeted for 2004, 11 for 2005 and eight for 2006. All the hospitals in the trial were state-run, public hospitals.

## Methods

The research proposal was approved by the Research Ethics Committee of the Faculty of Health Sciences, University of Pretoria (No 16/2002).

Thirty-six hospitals were eligible to participate in the randomised trial to test the effectiveness of two face-to-face facilitation strategies. The hospitals were paired with respect to their level of care, their geographical location (urban or rural) and the annual number of births at each facility (which varied between 200 and 7600 births per year). One hospital in each pair was randomly allocated to Group A, the other to Group B, by spinning a coin. Group A received on-site facilitation and Group B off-site facilitation.

### Facilitation process

The facilitation process followed a very distinct pattern in all cases. Hospitals were invited to voluntarily participate in the outreach. The Chief Executive Officer (CEO) of each hospital was required to sign a commitment of participation. All hospitals sent a multi-professional task team of three to six delegates to an introductory workshop. The task teams consisted of different combinations of managers, doctors, midwives, nurses, dieticians, occupational therapists, speech therapists, physiotherapists and social workers. The choice of which delegates should attend the workshop was left to the managers of the participating hospitals. At this workshop the delegates received training in the theory and practice of KMC and participated in practical activities related to the implementation process. Each hospital received an implementation package and was informed about the outreach strategy to which it had been allocated. The duration of the introductory workshop in Mpumalanga was two days and in Gauteng only one day, as health workers were more familiar with KMC as a result of previous training workshops.

**Figure 1:**
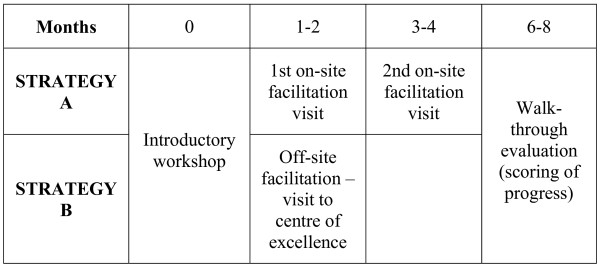
Process of implementation and facilitation.

'On-site' facilitation (Group A) entailed two site visits to hospitals, lasting two to three hours each. This started six to eight weeks after the introductory workshop and took place at four-weekly intervals. 'Off-site' facilitation (Group B) entailed a one- or two-day, 'hands-on' training workshop at hospitals identified as centres of excellence. This took place six to eight weeks after the introductory workshop. Three training centres, one in Gauteng and two in Mpumalanga, had well established KMC units and were available for this study. All three were regional hospitals with neonatal intensive care facilities. Figure 1 provides a graphic depiction of the process followed.

The same two resource persons conducted the introductory workshop and attended almost all of the facilitation sessions, one concentrating on clinical issues (EvR), the other on implementation issues (A-MB). The content of the workshop and facilitation sessions was built around an evidence-based workbook [10], which is part of the implementation package. An important aspect of the introductory workshop was the development of a plan of action by each hospital. This was photocopied and with each on-site or off-site visit participants were requested to give a presentation on their progress. At the end of each facilitation session, hospitals had to commit themselves to further steps in implementation, against which their progress could be measured at the next visit or at the assessment visit at the end.

### Outcome measures

**Figure 2:**
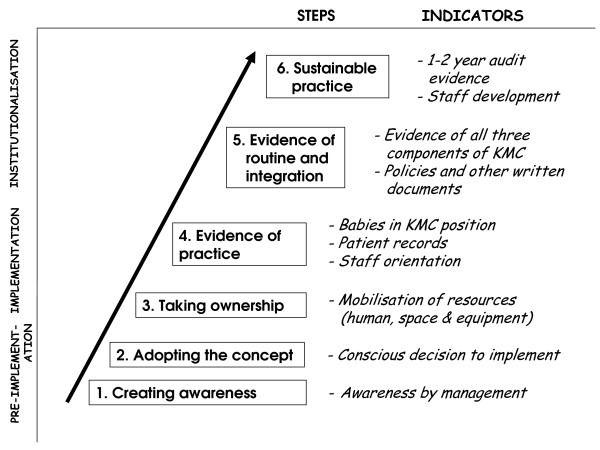
**The progress-monitoring model**. Adapted from Bergh *et al*. (2005) [11].

**Figure 3:**
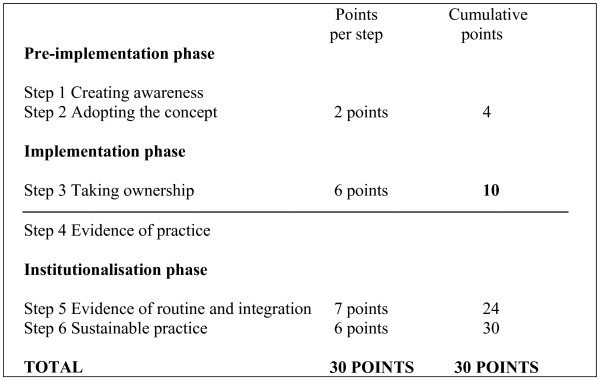
The scoring system for evaluating the implementation of KMC.

Six to eight months after the introductory workshop each hospital was visited and scored by means of a standardised instrument [11]. The evaluation team consisted of the two facilitators (A-MB & EvR), the provincial coordinators and other assessors trained in each province. The assessment instrument is based on a progress-monitoring model (see figure 2) that is divided into three phases: pre-implementation, implementation and institutionalisation. Each of these phases consists of two steps, starting with raising awareness and encouraging the hospital to take a conscious decision to implement, through to the hospital's taking ownership and showing evidence of practice, up to evidence of routine and institutionalised practice, with the ultimate goal being sustainable practice. Each step has specific indicators that are scored according to a weighted system [11]. The maximum score is 30 and hospitals scoring more than 10 out of 30 have reached the level of 'evidence of practice'. (See figure 3.)

## Results

Using the Wilcoxon paired ranked test, no significant difference was found in the effectiveness of the two outreach strategies (p = 0.633). The median score for the on-site facilitation group (A) was 16.52 (range 00.00–23.79) and for the off-site facilitation group (B) 14.76 (range 07.50–23.29). The mean scores were 15.03 and 14.87 respectively.

**Figure 4:**
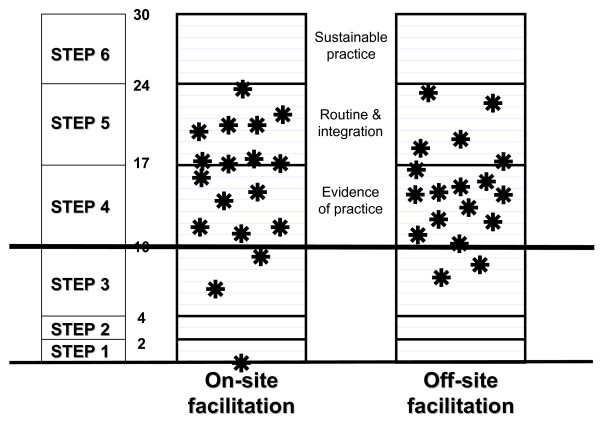
Distribution of scores of individual hospitals.

**Figure 5:**
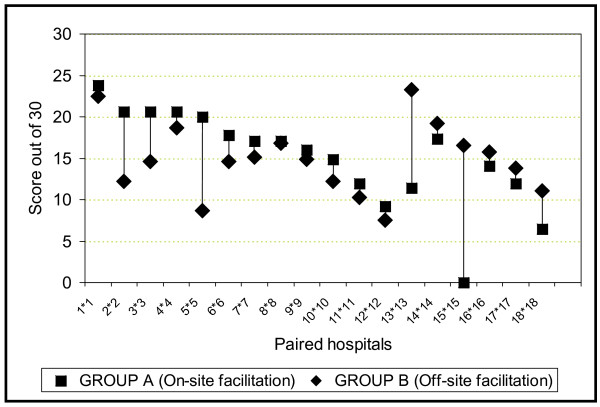
Performance of paired hospitals.

Thirty-one of the 36 hospitals in the trial reached at least the level of "evidence of practice" after six to eight months. One hospital in the on-site group had made no attempts at implementation and scored 0. Two other hospitals in this group scored <10 (6.42 and 9.21). In the off-site group two hospitals could not manage a score of >10 (7.50 and 8.67). Figure 4 provides a graphic depiction of the distribution of the scores of individual hospitals in the two groups, according to the steps of the progress-monitoring model (figures 2 and 3). Figure 5 shows the scores of the paired hospitals in relation to each other. There were no obvious features explaining differences between hospitals with on-site facilitation scoring better than their off-site pairs (pairs 1 to 12 in figure 5) nor between hospitals with off-site facilitation scoring better that their on-site pairs (pairs 13 to 18 in figure 5).

## Discussion

The implementation of KMC was successful and the scores of Group A (on-site facilitation) were remarkably similar to the on-site facilitation scores in the Ukugona trial [9]. This confirms the assumption that face-to-face facilitation is effective in the scaling up of new health care strategies.

Secondly, the finding in this study indicates that it is not crucial whether the face-to-face facilitation takes place at a centre of excellence or at the hospital where the new programme is to be implemented. This was surprising, as communication with peers created the expectation that off-site training would be less effective. However, in this programme there were certain aspects common to both implementation strategies, namely: the CEO of the hospital had to give a signed undertaking to implement the programme; a multidisciplinary team of health workers was involved; the same respected resource persons were responsible for the facilitation at, interaction with and feedback to all hospitals; and the team had to commit themselves to perform certain tasks by the time of the progress visit. It is possible that these aspects were more important than the actual venue of the face-to-face education. The implementation package contained all the information needed to implement KMC, as well as a workbook that, if followed, took the health workers through the implementation process step by step. The relative importance of these other factors still needs to be tested.

Five hospitals, three in Group A and two in Group B, failed to achieve evidence of practice. The one that failed completely to initiate the new intervention was a small hospital close to a busy highway, where health care staff was responsible for comprehensive services. Because of the workload, staff shortages and administrative constraints, they showed evidence of low morale. The KMC implementation team leader also left the service one month after the introductory workshop.

The weakness at all five hospitals that did not manage to implement KMC was a lack of sufficient opinion leaders who were convinced of the value of the programme. Subsequently no KMC protocols or guidelines were developed at these facilities. At some of the hospitals there was also reluctance by management to allocate a dedicated space where mothers could practise KMC 24 hours per day or to rearrange nursing staff allocations to include supervision for KMC. The drivers of the implementation process were often young enthusiastic health workers doing their obligatory community service year. They are usually replaced by new community service health workers each year. Key role players were either not involved in or not committed to the implementation process and this resulted in failure to sustain the practice. Two of the hospitals also had a history of trying to implement KMC, but being unable to sustain it.

Successful initiation of implementation does not mean that the KMC programme will be sustained. Factors such as staff turnover, a policy of staff rotations through different departments, the ability to orientate new staff, and enthusiasm for the process are the key factors.

In any scaling-up programme that is accompanied by education and training, health administrators have to decide which venue for face-to-face facilitation is most feasible and accessible. Cost will be a major deciding factor. Travel, accommodation, and all health workers' and facilitators' time away from work will need to be taken into account when calculating the costs, which is the subject of another investigation. The choice of strategy may furthermore depend on whether a new programme has to be implemented or whether the outreach is aimed at the quality improvement of existing practices. Another factor to consider is the scope of scaling up, which includes the number of sites where the new intervention or quality improvement programme would have to be implemented. For example, when 3000 primary health care clinics are targeted, other types of strategies may be required than in the case of an outreach targeting 36 hospitals as in this study.

## Conclusion

Our first trial illustrated that the implementation of a new health care intervention on a provincial scale was best achieved through a carefully designed educational package, combined with face-to-face facilitation by respected resource persons. This study demonstrated that the site of facilitation, either on site or at a centre of excellence, did not influence the ability of a hospital to implement KMC. The choice of outreach strategy could therefore be guided by local circumstances, cost and the availability of skilled facilitators.

As effective implementation strategies are costly, trade-offs may need to be made between educational effectiveness and cost benefits. This could be done by categorising hospitals in terms of ability to function without additional support and then deciding on differential strategies, according to each health care facility's capacity to implement a new health care intervention.

The results of testing the effectiveness of different outreach strategies could also inform policy decisions with regard to different kinds of roll-out or scaling-up programmes implemented by provincial and national health authorities.

## Competing interests

The authors declare that they have no competing interests.

## Authors' contributions

A-MB and RCP were involved in the original design of the research. A-MB and EvR were responsible for the facilitation of the implementation process and for data collection. A-MB did the data capturing and scoring of hospitals, whereas RCP did the statistical analyses. All three authors contributed to the drafting and revision of the manuscript.
